# Live-Cell-Based Assay Outperforms Fixed Assay in MOGAD Diagnosis: A Retrospective Validation Against the 2023 International Criteria

**DOI:** 10.3390/diagnostics16010157

**Published:** 2026-01-04

**Authors:** Anna Zhou, Weihua Zhang, Ji Zhou, Changhong Ren, Ke Zhan, Wenhan Li, Hui Xiong, Xiaotun Ren

**Affiliations:** 1 Department of Neurology, Beijing Children’s Hospital, Capital Medical University, National Centre for Children’s Health, Beijing 100045, China; zhouanna90@sina.com (A.Z.); 15811076790@163.com (W.Z.); zhoujibdek@163.com (J.Z.); libraren@yech.net (C.R.); 2 V-Medical Laboratory Co., Ltd., Hangzhou 311122, China; ke.zhan@ivydx.com (K.Z.); wenhan.li@ivydx.com (W.L.); 3 Xinjiang Hospital of Beijing Children’s Hospital, Urumqi 830054, China

**Keywords:** MOGAD, Live-CBA, Fixed-CBA, accuracy

## Abstract

**Background and Objective**: Myelin oligodendrocyte glycoprotein (MOG) antibody-associated disease (MOGAD) is a significant component of demyelinating diseases in pediatric populations. Recently, diagnostic criteria for MOGAD were established. This study aims to evaluate and compare the diagnostic efficacy of the fixed-cell-based assay (Fixed-CBA) and the live cell-based assay (Live-CBA) in patients who meet the 2023 clinical diagnostic criteria for MOGAD. **Methods**: This retrospective study included patients suspected of having MOGAD who were enrolled between June 2023 and June 2024. Patients were selected based on the “core clinical demyelinating events” outlined in the 2023 proposed criteria of the International MOGAD Panel. Patients with multiple sclerosis (MS), neuromyelitis optica spectrum disorders (NMOSD) with aquaporin-4 antibody-positive (AQP4-Abs-positive), and non-central nervous system (non-CNS) inflammatory diseases were chosen as controls. Serum samples were simultaneously tested for MOG-Abs using Fixed-CBA and Live-CBA. **Results**: A total of 86 patients were enrolled in the study: 52 in the suspected MOGAD group and 34 in the control group. Out of these patients studied, 16 presented with optic neuritis (ON), 5 with myelitis, 8 with acute disseminated encephalomyelitis (ADEM), and 7 with cortical encephalitis. Sixteen patients could not be classified by clinical phenotype. The highest MOG-Ab positivity rate was among patients with cortical encephalitis [85.7% (Live-CBA)/71.4% (Fixed-CBA)]. Both assays identified 22 positive samples, with Fixed-CBA and Live-CBA sensitivities at 44.2% and 55.8%, respectively, and a specificity of 97%. Of the patients suspected of having MOGAD, 19 cases were confirmed using the Fixed-CBA, while 28 cases were confirmed using the Live-CBA. This resulted in an upgrade in diagnostic classification for nine cases. This led to a diagnostic reclassification in nine cases. **Conclusions**: Both the Fixed-CBA and Live-CBA were associated with higher sensitivity for patients selected based on the 2023 MOGAD clinical diagnostic criteria. The Live-CBA exhibited an 11.6% increase in sensitivity, contributing to a 17.3% (9/52) enhancement in clinical diagnostic accuracy.

## 1. Introduction

The myelin oligodendrocyte glycoprotein (MOG) antibody-associated disease (MOGAD) is a prevalent autoimmune neurological disorder in pediatric populations. It is diagnosed through clinical presentation, imaging studies, and the detection of anti-MOG antibodies (MOG-Abs) [1]. The cell-based assay (CBA) utilizing full-length MOG is the recommended gold standard for detecting MOG-Abs [2], including both fixed-cell-based assay (Fixed-CBA) and the live cell-based assay (Live-CBA), although some studies have been conducted using flow cytometry [3]. In 2023, the International MOGAD Panel published the MOGAD diagnostic criteria in The Lancet Neurology. These criteria play a crucial role in diagnosing MOGAD. The sensitivity and specificity of these criteria are essential for an accurate diagnosis, and the criteria proposed by the panel have been validated in clinical practice [4,5,6]. Currently, Fixed-CBA are widely used in clinical settings by centers worldwide. However, the 2023 MOGAD criteria recommend the Live-CBA for detecting MOG-Abs [6]. Studies have shown that the positive predictive values (PPVs) associated with Live-CBA are higher than those of Fixed-CBA [7]. PPVs in children is higher than in adults [8]. Unfortunately, most relevant data comes from laboratory settings and has limited clinical application, especially in pediatric populations. Currently, most experts do not recommend MOG-Abs testing as a universal screening program, emphasizing selective testing based on clinical phenotype [9]. This study aims to evaluate and compare the performance and diagnostic accuracy of MOG-Abs detection using Fixed-CBA and Live-CBA, according to the diagnostic criteria for MOGAD established in 2023. The findings aim to inform the development of a diagnostic protocol for MOGAD in pediatric populations.

## 2. Methods

### 2.1. Standard Protocol Approvals, Registrations, and Patient Consents

All patients and their guardians who participated in this study provided informed consent for the use of their medical records for research purposes. The study received approval from the Ethics Committee of Beijing Children’s Hospital (approval number [2021]-E-098-Y).

### 2.2. Study Population and Controls

**Figure 1:**
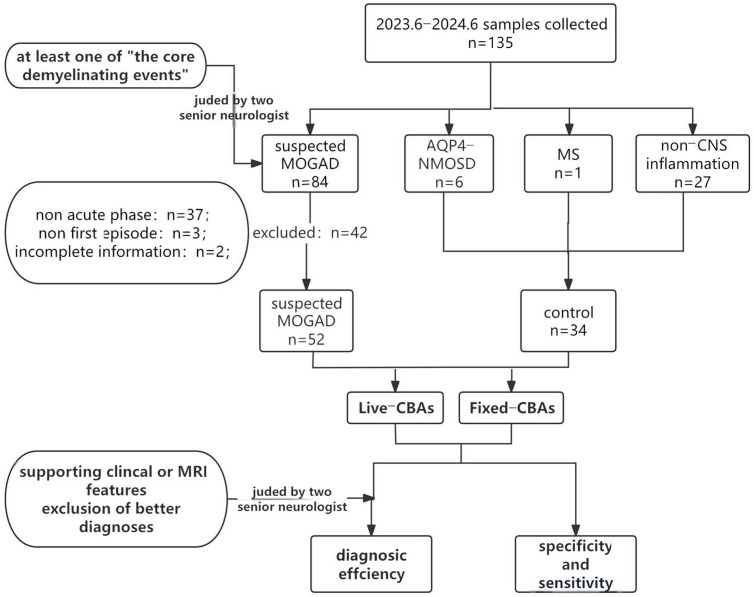
Study profile. Abbreviations: MOGAD = myelin oligodendrocyte glycoprotein antibody-associated disease; MS = multiple sclerosis; AQP4-NMOSD = neuromyelitis optica spectrum disorders with aquaporin-4 antibody; CNS = central nervous system; Fixed-CBA = fixed-cell-based assay; Live-CBA = live cell-based assay; MRI = Magnetic Resonance Imaging.

We conducted a retrospective review of the clinical records of patients with suspected demyelinating diseases who were referred to Beijing Children’s Hospital between June 2023 and June 2024. Two senior neurologists meticulously evaluated the clinical characteristics of all cases (Figure 1).

The study participants were patients suspected of having MOGAD and were referred to as the suspected MOGAD group. The inclusion criteria were as follows:

(1) participants must exhibit at least one of the core demyelination events outlined in the MOGAD criteria 2023 [6];
(2) participants must also not meet the diagnostic criteria for other central nervous system demyelinating diseases, such as multiple sclerosis (MS) or neuromyelitis optica spectrum disorder (NMOSD) with aquaporin-4 antibody (AQP4-Abs) positivity.

The exclusion criteria were as follows:

(1) serum samples obtained outside the acute disease stage (i.e., beyond one month after onset);
(2) incomplete data.

Patients diagnosed with MS, NMOSD with AQP4-Abs positivity, or non-inflammatory neurological disorders were selected to comprise the control group.

Patients diagnosed with MS, NMOSD with AQP4-Abs positivity, or non-inflammatory neurological disorders were selected to comprise the control group. We deliberately selected a composite control group comprising AQP4+ NMOSD, MS, and non-inflammatory conditions to reflect the real-world differential diagnosis of MOGAD in a pediatric neuroinflammatory clinic. This design allows us to estimate the clinical specificity of both assays against the precise spectrum of conditions from which MOGAD must be distinguished, thereby providing a more clinically relevant specificity estimate than a control group of only healthy individuals.

We conducted the detection of MOG-Abs in the patient’s serum concurrently using both Fixed-CBA and Live-CBA. This allowed us to calculate the sensitivity and specificity of each method. We then compared the diagnostic accuracy in accordance with the MOGAD criteria. To elucidate the relationship between clinical phenotypes and MOG-Abs positivity prevalence, we categorized patients’ phenotypes into the following groups: optic neuritis (ON) with or without intracranial lesions; myelitis; acute disseminated encephalomyelitis (ADEM); cortical encephalitis; and an unclassifiable group, characterized by isolated or multiple lesions in the brainstem and/or cerebrum.

### 2.3. MOG-IgG Assays

MOG antibody detection was performed using a Fixed-CBA with commercially available reagent kits (EUROIMMUN, Lübeck, Germany, FA 1156-1010-50) according to the manufacturer’s protocol. The assay used slides coated with fixed HEK-293 cells that had been transfected with a full-length human MOG construct. Patient sera were diluted at a ratio of 1:10 and incubated with the prepared slides. Then, anti-MOG IgG was identified using fluorescein isothiocyanate (FITC)-labeled anti-human IgG. Fluorescence intensity was assessed using a fluorescence microscope.

Live-CBA for MOG antibody detection: Human embryonic kidney 293 (HEK-293) cells expressing the full-length human MOG protein were cultured in a suitable growth medium. After removing excess culture medium, 50 μL of the diluted test serum sample (dilution ratio 1:10) in complete culture medium containing 10% fetal bovine serum was added. The cells were then incubated in a CO_2_-enriched environment for one hour. After incubation, the serum sample was removed, and the cells were washed with a PBS-Tween buffer. A fixative solution was then applied to stabilize the cellular structures at room temperature. After a 10 min fixation period, the fixative solution was removed, and the cells were washed again with Phosphate-Buffered Saline (PBS). The same aliquot from each sample was tested in parallel using both the Live- and Fixed-CBA with titers indicated.

For each testing batch, internal quality control was performed. Both negative and low-positive control samples were randomly placed and tested in parallel with clinical samples. Strict criteria were applied for result interpretation, and detailed records were kept to ensure full traceability of all results, in accordance with laboratory quality management system requirements for immunoassays.

For both Live- and Fixed-CBA, all serum samples were evaluated independently by two experienced observers who were blinded to the clinical data and the result of the other assay. Any discrepant results were adjudicated by a third senior observer. The inter-observer agreement in result interpretation was excellent, with a Cohen’s kappa of 0.95 for Live-CBA and 0.97 for Fixed-CBA.

### 2.4. Statistical Analysis

Statistical analyses were conducted using IBM SPSS Statistics for Windows, Version 26.0 (IBM Corporation, Armonk, NY, USA). Categorical variables were presented as frequencies and percentages. Fisher’s exact test was used to evaluate the MOG-Abs positivity rate. A *p*-value of less than 0.05 was considered statistically significant.

## 3. Results

A total of 52 patients were enrolled in the suspected MOGAD group, 27 of whom were male. The mean age of the cohort was 8.2 ± 2.9 years. Based on the core demyelination events outlined in the “International MOGAD Panel Proposed Criteria” [5], the distribution of demyelination events was as follows: Sixteen cases presented with ON, five cases presented with myelitis, eight cases presented with ADEM, thirteen cases presented with brainstem or cerebellar deficits, and seven cases presented with cerebral cortical encephalitis, five of which involved seizures. Among the aforementioned 52 patients, a total of 30 cases met the criteria of “cerebral monofocal or polyfocal deficits”. A control group consisting of 34 non-MOGAD patients was selected for the study. This group included six cases of NMOSD with AQP4-Abs positivity, one case of MS, 21 cases of functional headache, four cases of psychological disorders, one case of metabolic disease, and one case of esotropia. The cohort comprised 21 males, with a mean age of 9.8 (±2.9) years.

**Figure 2:**
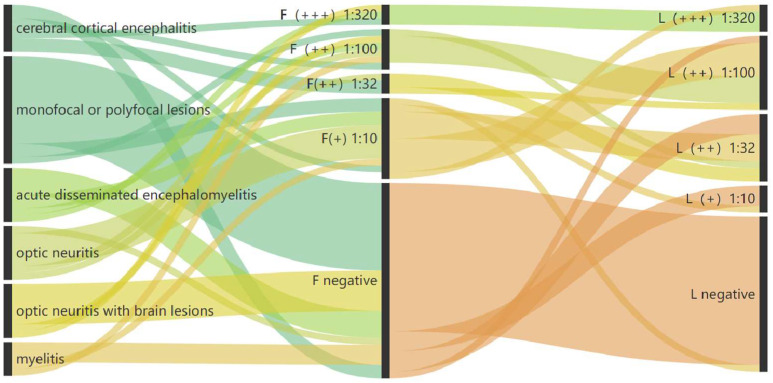
The results of Fixed-CBAs and Live-CBAs. (+): Low positive MOG antibody titer (1:10). (++): Moderate positive MOG antibody titer (1:32, 1:100). (+++): High positive MOG antibody titer (1:320). negative: no MOG antibodies detected.

We conducted parallel detection of MOG-Abs using Fixed-CBA and Live-CBA on serum samples. The results showed that 24 samples tested positive for MOG-Abs using Fixed-CBA, eight of which exhibited high titers (≥1:100) [1:10 (*n* = 13), 1:32 (*n* = 3), 1:100 (*n* = 5), 1:320 (*n* = 3)]. In contrast, MOG-Ab positivity was observed in 30 samples using Live-CBA, 15 of which displayed high titers [1:10 (*n* = 5), 1:32 (*n* = 10), 1:100 (*n* = 11), 1:320 (*n* = 4)] (Figure 2). Twenty-two samples demonstrated concordant positivity across both assays. Live-CBA had a sensitivity of 55.8%, surpassing Fixed-CBA’s sensitivity of 44.2%. The specificity of the two assays was comparable, as detailed in Table 1.

**Figure 3:**
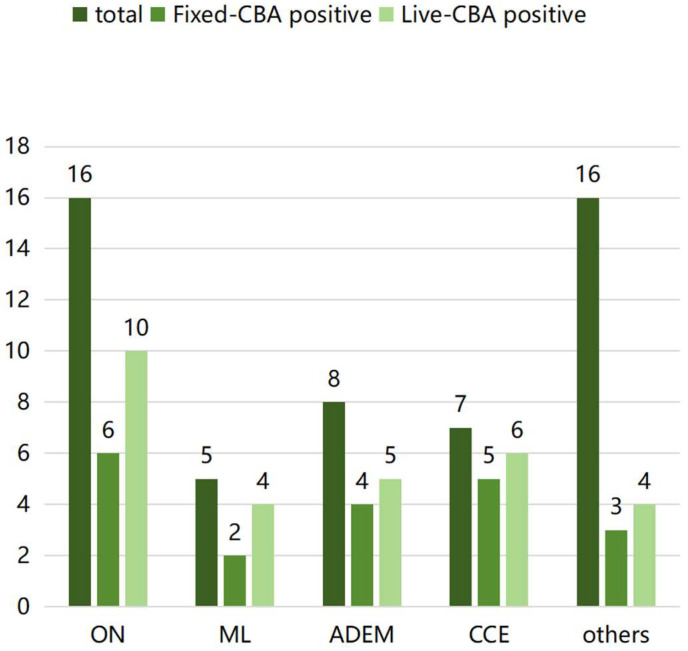
The frequency of MOG-Abs positivity on the Fixed-CBAs and Live-CBAs. Abbreviations: ON = optic neuritis; ML = myelitis; ADEM = acute disseminated encephalomyelitis; CCE = cerebral cortical encephalitis; others = unclassified phenotype.

According to the clinical phenotype-based classification, 16 patients were identified as having ON, either with or without intracranial lesions. Five patients were classified as having myelitis, eight as having ADEM, and seven as having cortical encephalitis. Sixteen cases did not conform to these criteria and were characterized by monofocal or polyfocal brain lesions. The highest proportion of MOG-Abs was detected in the cortical encephalitis phenotype, with positivity rates of 71.4% and 85.7% on the Fixed-CBA and Live-CBA, respectively. Patients categorized under the four recognized phenotypes exhibited positivity rates of 47.2% (Fixed-CBA) and 69.4% (Live-CBA), which are higher compared to those with other unclassified (Supplemental Table S1) phenotypes (Fixed-CBA: 18.7%, χ^2^ = 6.399, *p* = 0.171; Live-CBA: 25%, χ^2^ = 9.862, *p* = 0.036) (Figure 3).

In our study, patients were initially enrolled based on “core demyelination events”. We then incorporated “positive MOG-IgG test” results and “supporting clinical or MRI features” [5] for a more comprehensive evaluation. This allowed us to compare the diagnostic accuracy of the two distinct assays. The results showed that, of the suspected MOGAD cohort, 19 cases were diagnosed as definite MOGAD using Fixed-CBA, yielding a diagnosis rate of 36.6% (19/52). An additional nine patients were diagnosed using Live-CBA (Table 2). Two patients with lateral neuritis had their diagnoses upgraded due to increased MOG-Abs titers, while the remaining seven patients, who tested negative with Fixed-CBA, tested positive with Live-CBA. The diagnosis rate with Live-CBA was 53.8% (28/52), representing a 17.3% (9/52) increase in MOGAD diagnoses (Figure 2). All nine cases uniquely identified by Live-CBA were positive at a moderate to high titer (≥1:10) and were tested on serum drawn during an acute clinical attack. Repeat testing on a separate aliquot confirmed positivity in nine cases.

## 4. Discussion

The Fixed-CBA is an experimental approach in which cells are transfected to express the full-length MOG protein, are fixed, and used as a substrate to detect anti-MOG IgG antibodies. The fixation process preserves the cells’ intrinsic morphology and structural integrity, preventing dissolution, destruction, and autolysis resulting from lysosomal enzyme activity, while maintaining the stability of various protein components, facilitating long-term preservation. However, fixation may affect antigen epitopes, which could interfere with the binding of anti-MOG IgG in serum to MOG protein. This interference may result in either false positive or false negative outcomes.

Conversely, the Live-CBA uses cultured cells to detect antibodies, which optimally preserves the native conformation of the MOG protein. Consequently, CBA demonstrates greater sensitivity in detection than Fixed-CBA [7,10,11]. However, no randomized controlled trials have been conducted to compare the two assays, which precludes definitive conclusions regarding their relative efficacy. This study retrospectively enrolled patients and compared the assays in a clinical setting to establish a basis for future prospective research.

This study found that the specificity of the two assays was comparable, with no observed difference in PPV. However, the sensitivity was higher than previously reported [7]. This discrepancy may be due to the different inclusion criteria of the two studies. Previous studies have reported a sensitivity range of 23.1% to 27.5% for MOG-Ab detection [7], which is lower than the sensitivity observed in our study. Our patient cohort was selected based on the 2023 MOGAD criteria, which may explain the notable increase in sensitivity. Additionally, the PPV of both methods in this study was higher than that reported in other literature. This is due not only to differences in inclusion criteria, but also to the fact that the pediatric patients we selected had a higher PPV for MOG-Ab testing than adults [8]. The “International MOGAD Panel Proposed Criteria”, refined in 2023, improved upon earlier versions by enhancing the diagnostic parameters and demonstrating high sensitivity and specificity [5,12]. Professor Dalmau emphasized that “the combination of high clinical interest and easy access to the antibody-detection tests creates the perfect conditions for the emergence of potentially serious problems, one of which is the indiscriminate testing of antibodies” [13], a perspective with which we concur. Indiscriminate antibody detection not only exacerbates the economic burden but also interferes with accurate diagnosis and results in inappropriate treatment. The 2023 MOGAD criteria offer practical guidelines for neurologists to select appropriate objectives based on clinical information, and they should be widely recommended and applied. Ultimately, judicious patient selection based on diagnostic criteria is essential for enhancing diagnostic efficiency.

MOGAD is recognized as the predominant demyelinating disease in pediatric populations, accounting for 30–50% of cases [14]. For this study, patients were rigorously selected based on the 2023 MOGAD criteria. The findings indicated that MOGAD constituted between 36.6% and 55.8% of cases, depending on whether Fixed-CBA or Live-CBA was used, which aligns with data from other research centers [15]. The most frequently observed phenotype in this cohort was ON, with or without accompanying brain lesions, followed by ADEM and cortical encephalitis. The positivity rate of MOG-Abs was highest among patients with cortical encephalitis, followed by those with myelitis and ADEM (Figure 3). Conversely, the detection rate of MOG-Abs in samples from unclassified patients was the lowest, suggesting a phenotypic preference for MOGAD in pediatric patients. The clinical phenotype was the primary indicator for physicians when diagnosing these diseases. Currently, the classical phenotypes of MOGAD are widely acknowledged. However, further in-depth research is necessary to better understand the non-classical phenotypes. The 2023 MOGAD criteria not only facilitate the diagnosis of classical phenotypes, but also enable the identification of certain non-classical phenotypes, demonstrating considerable practical applicability.

The high proportion of ‘unclassified’ phenotypes (Supplemental Table S1) in our cohort, which showed markedly lower seropositivity, highlights the challenge of MOGAD diagnosis in clinically atypical cases. This heterogeneity may introduce spectrum bias, as our cohort‘s assay performance is evaluated across the full clinical spectrum of suspected MOGAD, not just classic phenotypes. The particularly low positivity rate in this subgroup reinforces that in patients with non-classical presentations, a negative MOG-IgG result should be interpreted with caution, and the diagnosis must rely more heavily on comprehensive clinical and radiological assessment.

Crucially, the absolute sensitivity of 55.8% for even the more sensitive Live-CBA provides a critical benchmark for clinicians. This figure underscores a fundamental principle applicable to all suspected MOGAD cases: a single negative serum antibody test cannot rule out the disease. Therefore, the diagnosis of MOGAD remains rooted in clinical-radiological syndromic assessment, with antibody testing serving as a supportive, but not definitive, discriminant. In practice, for patients with a high clinical suspicion for MOGAD despite seronegativity, strategies such as repeat testing during a future attack or consideration of cerebrospinal fluid MOG-IgG testing should be considered to mitigate the risk of false-negative diagnoses [16,17].

In pediatric MOGAD, encephalitis is one of the primary phenotypes [18], with some overlapping phenotypes to those in the 2023 diagnostic criteria. However, we found that some rare phenotypes, such as aseptic meningitis [19,20], tumefactive demyelination [21], were not included in the diagnostic criteria. Seven patients presenting with isolated aseptic meningitis were excluded from this study following the filtering process, three of whom tested positive for MOG-Abs. These results suggest that additional phenotypes should be incorporated into future diagnostic criteria to enhance the sophistication of diagnostic systems [22].

Conversely, detecting MOG-Abs is crucial for diagnosis. The cell-based assay (CBA) is regarded as the “gold standard” for MOG-IgG detection [23] and maintains a definitive role in diagnosing MOGAD. Our research indicates that Live-CBA enhances diagnostic accuracy compared to Fixed-CBA. In this study, nine additional patients were diagnosed with MOGAD using Live-CBA. These patients presented with various phenotypes. The most common phenotype observed was isolated ON without intracranial lesions (Table 2), which is consistent with previous reports [24,25]. Different types of central nervous system inflammatory demyelinating diseases (CIDD) require distinct treatment protocols [26,27,28]. For example, regarding corticosteroid therapy, a daily dose of 0.16 mg/kg of prednisone administered for at least three months during the initial MOGAD episode in children has been shown to delay the time to the first relapse [29]. Timely diagnosis is crucial in mitigating the short-term recurrence of symptoms in patients with MOGAD. Furthermore, numerous other factors influence the detection of MOG-Abs; several of these factors currently lack a consensus solution. These factors include the timing selected for antibody detection and the differentiation of MOG-IgG subclasses. Additional research is necessary to enhance international standardization of clinical care [7,10].

Our research demonstrated that the sensitivity and diagnostic accuracy increased according to the 2023 MOGAD diagnostic criteria when using Live-CBA. However, the cells used in Live-CBA have a short storage lifespan and require strict laboratory conditions, stable reagents, repeatability, and advanced inspection techniques. Consequently, these assays are feasible only in specialized laboratory settings. Furthermore, there are significant variations in the production and inspection processes of Live-CBA across different laboratories, which makes achieving standardized uniformity within the industry challenging [30]. Therefore, Live-CBA is currently unsuitable for use as a primary method for detecting MOG-Abs.

Future randomized controlled trials (RCTs) are necessary to ascertain whether Live-CBA offers a definitive advantage over Fixed-CBA. Due to existing technical disparities, it is currently inadvisable to mandate the use of Live-CBA for detecting MOG-Abs in all patients. Initially, employing Fixed-CBA is advisable. For patients with classic phenotypes, if MOGAD cannot be diagnosed due to negativity or low titers on Fixed-CBA, or for patients with nonclassical phenotypes where Fixed-CBA indicates low titers, it is recommended that Live-CBA be performed subsequently in certified laboratories. It is crucial to emphasize that these tests must be conducted in professional laboratories to ensure accurate results.

It is important to note that the observed difference in sensitivity between the assays may be influenced by factors inherent to our retrospective study design. These include the timing of serum sampling relative to clinical onset and relapse, the distribution of clinical phenotypes within our cohort, and the potential for spectrum bias. While our findings robustly demonstrate an association between Live-CBA and increased MOG-IgG detection in this real-world pediatric cohort, future prospective studies with standardized sampling protocols are needed to definitively establish causal inference regarding assay performance.

A significant limitation of this study is its observational design. As it was a retrospective analysis based on clinical data, it may be subject to bias. Future research should incorporate prospective randomized controlled trials to mitigate this limitation. Additionally, the study was conducted exclusively within our laboratory, limiting the applicability of the findings to internal use. To strengthen the evidence base, future investigations should involve standardized research across multiple centers.

Despite the current practical limitations of Live-CBA—including restricted availability and lack of standardization—our data suggest that its incorporation into diagnostic pathways, where available and performed in experienced centers, can significantly reduce false-negative diagnoses. We therefore recommend a tiered approach: in cases of high clinical suspicion for MOGAD but a negative Fixed-CBA, referral of samples to a specialized center for Live-CBA testing should be strongly considered. This strategy balances the need for heightened diagnostic sensitivity with the practical constraints of assay availability.

## 5. Conclusions

Both the Fixed-CBA and Live-CBA demonstrated high specificity for patients selected based on the MOGAD clinical diagnostic criteria 2023. The Live-CBA increased sensitivity by 11.6% and contributed to a 17.3% (9/52) improvement in clinical diagnostic accuracy.

## Figures and Tables

**Table 1:** Comparison between Fixed-CBA and Live-CBA in our study. Abbreviations: NPV = negative predictive values; PPV = positive predictive values.

	Sensitivity	Specificity	PPV	NPV
Fix–CBA	44.2%	97.1%	95.8%	53.2%
Live–CBA	55.8%	97.1%	96.7%	58.9%

**Table 2:** Information of the 9 patients who were additionally diagnosed as MOGAD using the Live-CBA. Abbreviations: ON = optic neuritis; ML = myelitis; ADEM = acute disseminated encephalomyelitis; BCD = brainstem or cerebellar deficit; CD = cerebral monofocal or polyfocal deficits; CCE = cerebral cortical encephalitis often with seizures; + presence of the indicated clinical feature; - absence of the indicated clinical feature or negative result on the MOG antibody test.

Patient No.	Age (y)	Core Demyelination Events						MOG-Abs Test		Supporting Clinical or MRI Features
		ON	ML	ADEM	BCD	CD	CCE	Fix-CBA	Live-CBA	
2	7.5	+ (left)	−	−	−	−	−	1:10	1:320	−
3	7.9	−	−	−	−	+	+	−	1:32	+
5	6.5	+ (right)	−	−	−	−	−	1:10	1:100	−
27	6.8	+ (bilateral)	−	−	−	+	−	−	1:100	+
29	11.5	−	−	−	+	+	−	−	1:32	+
31	11.8	−	+	−	+	−	−	−	1:10	+
46	10.5	+ (bilateral)	−	−	−	−	−	−	1:10	+
48	9.1	−	−	+	−	+	−	−	1:10	+
49	6.9	−	+	−	−	−	−	−	1:32	+

## Data Availability

The original contributions presented in this study are included in the article/Supplementary Material. Further inquiries can be directed to the corresponding author.

## Supplementary Materials

The following supporting information can be downloaded at: https://www.mdpi.com/article/10.3390/diagnostics16010157/s1, Table S1. Clinical, Radiological and Serological Features of the 16 Patients with Unclassified MOGAD Phenotypes.
