# Supplementary material for: Live-Cell-Based Assay Outperforms Fixed Assay in MOGAD Diagnosis: A Retrospective Validation Against the 2023 International Criteria
Source: Diagnostics (Basel). 2026 Jan 4;16(1):157. doi: 10.3390/diagnostics16010157 (PMC12785690; doi:10.3390/diagnostics16010157)
Supplement: Supplementary file 1 [file diagnostics-16-00157-s001.zip › diagnostics-4002505-supplementary.pdf]

Supplemental Table S1. Clinical, Radiological and Serological Features of the 16 Patients with Unclassified MOGAD Phenotypes.

Patient No.	Age（y）	Sex	Clinical features	MRI	F-CBA result	L-CBA result
6	4.2	M	strabismus, ataxia	multiple lesions in brainstem, cerebellum, cerebrum, and spinal cord	-	-
8	12.1	M	headache, limp	isolated lesion in brainstem	+1:10	1:10
10	6.5	M	fever, headache	isolated lesion in cerebrum	-	-
19	9.8	M	fever, headache	isolated lesion in cerebrum	++1:100	++ 1:100
24	10.3	F	vomiting, seizures	multiple lesions in cerebrum	+1:10	++ 1:32
29	11.1	F	fever, dizziness, headache, diplopia	isolated lesion in brainstem	-	++ 1:32
32	11.7	M	fever, headache	multiple lesions in cerebrum	-	-
33	6.4	F	fever, seizure	multiple lesions in cerebrum	-	-
34	4.0	M	fever, seizure, drowsiness	multiple lesions in cerebrum	-	-
37	6.0	F	drowsiness, gait unsteadiness	multiple lesions in brainstem, cerebrum	-	-
38	13.5	F	fever, seizures, impaired consciousness	multiple lesions in cerebrum	-	-
39	11.2	M	limb weakness	multiple lesions in cerebrum	-	-
40	12.2	F	dizziness, vomiting, limb weakness	multiple lesions in brainstem, cerebellum, cerebrum, and spinal cord	-	-
41	8.8	M	fever, seizures, impaired consciousness	isolated lesion in cerebrum	-	-
42	8.9	M	fever, seizures	multiple lesions in cerebrum	-	-
52	4.8	M	seizures, personality change, hypersomnia	multiple lesions in cerebrum	-	-

Abbreviations: F-CBA, fixed cell-based assay; L-CBA, live cell-based assay; MRI, magnetic resonance imaging.
